# GLP-1 receptor agonists as potential disease-modifying agents in Parkinson’s and Alzheimer’s disease: mechanisms, clinical trial evidence, and the neuroprotection horizon

**DOI:** 10.3389/fnagi.2026.1969490

**Published:** 2026-10-07

**Authors:** Mohamed Alsharif, Omran Shrebaty, Mohammad Ammar Aloudah, Mayar Alqahtani, Raghad Saud Aloubili, Marafi Jammaa Ahmed, Bana Albani, Ahmad El Bana

**Affiliations:** 1 College of Medicine, Alfaisal University, Riyadh, Saudi Arabia; 2 College of Medicine, Sulaiman Alrajhi University, Al Bukayriyah, Saudi Arabia; 3 College of Medicine, Vision Colleges, Riyadh, Saudi Arabia; 4 College of Medicine, Imam Mohammad Ibn Saud Islamic University (IMSIU), Riyadh, Saudi Arabia; 5 Faculty of Medicine, University of Bahri, Khartoum, Sudan; 6 College of Medicine, AlMaarefa University, Riyadh, Saudi Arabia; 7 Faculty of Medicine, Al Azhar University, Cairo, Egypt

**Keywords:** Alzheimer’s disease, exenatide, GLP-1 receptor agonists, neurodegeneration, neuroprotection, Parkinson’s disease, semaglutide

## Abstract

Glucagon-like peptide-1 receptor agonists (GLP-1 RAs), originally developed as antidiabetic agents, have emerged as candidate disease-modifying therapies for Parkinson’s disease (PD) and Alzheimer’s disease (AD), reflecting the widespread expression of GLP-1 receptors across neurons, glia, and the cerebral vasculature. This narrative review synthesizes mechanistic, clinical trial, and biomarker evidence for GLP-1 RAs in PD and AD, based on a literature search of PubMed, Embase, Scopus, and Web of Science, with ClinicalTrials.gov searched separately for trial protocols and results, through May 2026. Preclinical data implicate GLP-1 receptor agonism in anti-neuroinflammatory signaling, mitochondrial preservation, autophagy and proteostasis enhancement, anti-apoptotic effects, synaptic plasticity, cerebrovascular protection, and glymphatic clearance. Clinical evidence in PD is mixed and agent- and formulation-dependent: the phase II Exenatide-PD trial reported a motor benefit that persisted after washout (∼3.5-point between-group difference in MDS-UPDRS Part III), and the phase II LIXIPARK trial (lixisenatide) reported a significant 3.08-point between-group difference (95% CI 0.86–5.30; *p* = 0.007) favoring lixisenatide, whereas the larger, longer phase III Exenatide-PD3 trial, using an extended-release weekly formulation, found no significant effect on the same endpoint (adjusted coefficient 0.92; 95% CI −1.56 to 3.39; *p* = 0.47); oral semaglutide is under investigation in the ongoing phase II MOST-ABLE trial. In AD, the phase IIb liraglutide (ELAD) trial did not achieve statistical benefit on either its primary imaging endpoint (cerebral glucose metabolic rate; *p* = 0.14) or on most secondary cognitive measures, and the phase III semaglutide trials EVOKE and EVOKE+ did not meet their primary CDR-SB endpoint (estimated difference −0.08, 95% CI −0.35 to 0.20, *p* = 0.57 in EVOKE; 0.10, 95% CI −0.17 to 0.38, *p* = 0.46 in EVOKE+), despite biomarker signals including reductions in cerebrospinal fluid tau species and inflammatory markers. Comparative pharmacology reveals substantial heterogeneity in central nervous system penetration, receptor engagement, and systemic bioavailability across exenatide, liraglutide, semaglutide, and the imbalanced GLP-1/GIP co-agonist tirzepatide, complicating cross-agent extrapolation. Persistent challenges include separating direct neuroprotection from indirect metabolic and cardiovascular benefit, limited characterization of brain drug exposure, trial durations that may be short relative to the expected latency of a disease-modifying effect, and the absence of validated response biomarkers. We conclude that although the mechanistic and epidemiological rationale for GLP-1 RAs in neurodegeneration remains biologically plausible, current trial evidence does not yet support their use outside clinical trials, irrespective of comorbid diabetes status; future studies should prioritize earlier intervention, biomarker-guided patient selection, adequately powered subgroup analyses, and brain-penetrant formulations.

## 1 Introduction

Glucagon-like peptide-1 receptor agonists (GLP-1 RAs) entered clinical practice as antidiabetic agents engineered to mimic the incretin effect and improve glycaemic control in type 2 diabetes mellitus (T2DM). Over the past decade, this drug class has undergone an unanticipated repositioning: preclinical and early clinical evidence now frames GLP-1 RAs as pleiotropic neuroprotective candidates with disease-modifying potential in Parkinson’s disease (PD) and Alzheimer’s disease (AD), a shift driven largely by the recognition that the GLP-1 receptor (GLP-1R) is widely expressed in the central nervous system and engages pathways relevant to neuronal survival (Reich and Hölscher, 2022).

This repositioning has been reinforced by epidemiological signals. In a population-based cohort of Medicare beneficiaries with T2DM, new use of a GLP-1 RA was associated with a 23% lower risk of incident PD compared with new use of a dipeptidyl peptidase-4 inhibitor (DPP4i) (hazard ratio [HR] 0.77, 95% CI 0.63–0.95) (Tang et al., 2024). A parallel signal has emerged for dementia: in a longitudinal cohort of 109,778 individuals with T2DM, GLP-1 RA use was associated with a reduced incidence of dementia relative to non-use (adjusted HR 0.90, 95% CI 0.83–0.97) (Cheng et al., 2025). These associative findings, drawn from patients already being treated for metabolic disease, do not establish causation, but they provided an initial rationale for investigating GLP-1 RAs as standalone neuroprotective agents.

The biological plausibility of this rationale rests on the understanding that the brain is an insulin-sensitive organ, a concept that underpins the “type 3 diabetes” hypothesis of AD, which frames impaired central insulin signaling as a shared pathophysiological driver of amyloid-β accumulation, tau hyperphosphorylation, and neuroinflammation alongside the metabolic disturbances of T2DM (Michailidis et al., 2022).

The impetus for dedicated neurodegeneration trials came from a pooled analysis combining three randomized, double-blind cardiovascular outcome trials of semaglutide and liraglutide (15,820 patients) with a nationwide Danish registry cohort (120,054 patients), which found a lower dementia rate among GLP-1 RA-treated patients in both data sources (trial HR 0.47, 95% CI 0.25–0.86; registry HR 0.89, 95% CI 0.86–0.93 per year of exposure) (Nørgaard et al., 2022). The design paper for the subsequent EVOKE and EVOKE+ Alzheimer’s disease trials explicitly cites this accumulated preclinical, real-world, and cardiovascular-trial evidence base as the rationale for launching dedicated, disease-specific investigation (Cummings et al., 2025). Interest in repurposing was further reinforced when the SELECT trial showed that weekly subcutaneous semaglutide 2.4 mg reduced major adverse cardiovascular events by 20% in patients with pre-existing cardiovascular disease and overweight or obesity but without diabetes (hazard ratio 0.80, 95% CI 0.72–0.90), demonstrating a cardioprotective benefit independent of glycaemic status (Lincoff et al., 2023).

Building on this foundation, the present narrative review synthesizes mechanistic, clinical trial, and biomarker evidence evaluating GLP-1 RAs as disease-modifying agents in PD and AD, with attention to comparative pharmacology across agents, the biological plausibility of central nervous system (CNS) engagement, and the unresolved questions that currently limit translation into clinical practice. Unlike prior narrative reviews in this area, which have largely summarized mechanistic and epidemiological data in isolation, this review places the phase II–III trial programmes for both diseases alongside their biomarker sub-studies and comparative pharmacokinetic data, with the explicit aim of identifying why mechanistic promise has, to date, translated inconsistently – and disparately between PD and AD – into confirmed clinical benefit.

## 2 Methods

This narrative review followed the general framework recommended for narrative reviews in the biomedical literature, with an explicit search strategy and pre-specified eligibility criteria, while retaining the interpretive and synthesizing approach appropriate to a mechanistically oriented topic that spans preclinical, epidemiological, and phase II–III trial evidence.

### 2.1 Search strategy

A literature search was performed across PubMed, Embase, Scopus, and Web of Science for published mechanistic, epidemiological, and clinical trial literature, with a date limit extending to May 2026. ClinicalTrials.gov and, where relevant, non-US trial registries (e.g., the Japan Registry of Clinical Trials) were searched separately and in parallel to identify ongoing or completed trial protocols and results not yet indexed in the bibliographic databases. Search terms, combined using Boolean operators, included “GLP-1 receptor agonist Parkinson’s,” “semaglutide Alzheimer’s,” “liraglutide neuroprotection,” “exenatide Parkinson’s disease,” “GLP-1 neuroinflammation,” “tirzepatide neurodegeneration,” and “incretin brain insulin resistance.” A representative full search string, adapted across databases, was: (“GLP-1 receptor agonist*” OR semaglutide OR liraglutide OR exenatide OR lixisenatide OR tirzepatide) AND (Parkinson* OR Alzheimer* OR neurodegenerat* OR neuroprotect*). Reference lists of retrieved articles and recent systematic reviews were hand-searched to identify additional eligible studies, including trial protocols and conference abstracts reporting biomarker sub-studies not yet available as full manuscripts.

### 2.2 Eligibility criteria

Human randomized controlled trials, observational cohort studies, mechanistic preclinical studies, and publicly registered ongoing trial protocols were eligible for inclusion. Eligible records were limited to English-language, peer-reviewed publications or, for ongoing trials, publicly registered protocols on ClinicalTrials.gov or equivalent national registries. Priority was given to randomized controlled trials and large-scale registry or cohort studies when synthesizing clinical evidence, with preclinical and mechanistic studies used selectively to support biological plausibility rather than as primary evidence of clinical efficacy.

### 2.3 Scope and limitations of the approach

As a narrative rather than systematic review, this work does not apply formal risk-of-bias scoring or meta-analytic pooling, and no PRISMA-style flow diagram of records identified, screened, and excluded was generated; study quality is instead addressed qualitatively within each section, and the Discussion section explicitly weighs methodological limitations of the underlying trial literature. Readers seeking a fully quantified, systematic study-selection process are directed to existing systematic reviews and meta-analyses in this area, including a recent systematic review and meta-analysis of cognitive outcomes with liraglutide and exenatide in AD and mild cognitive impairment (Santos et al., 2026). Mechanistic findings are, in places, discussed collectively across the GLP-1 RA class for narrative clarity; as detailed in Section “7 Comparative pharmacology: exenatide vs. liraglutide vs. semaglutide vs. tirzepatide,” however, individual agents differ substantially in receptor selectivity, bioavailability, and blood–brain barrier permeability, and mechanistic or clinical findings from one agent should not be assumed to generalize to others without direct supporting evidence.

## 3 GLP-1 receptor biology in the central nervous system

GLP-1 receptors have been mapped in the brain to the hypothalamus, hippocampus (including the CA1 subfield), temporal and cerebral cortex, striatum, and substantia nigra, where they are expressed on tyrosine-hydroxylase-positive dopaminergic neurons; expression also extends to the locus coeruleus, amygdala, and several brainstem nuclei (Reich and Hölscher, 2022).

At the cellular level, GLP-1R is a class B G-protein-coupled receptor. Upon activation, it stimulates adenylate cyclase, raising intracellular cyclic AMP and activating protein kinase A (PKA) signaling. PKA-dependent signaling drives phosphorylation of the transcription factor CREB, which promotes transcription of brain-derived neurotrophic factor (BDNF) and thereby supports synaptic plasticity and neuronal survival. In parallel, GLP-1R activation engages the PI3K/Akt pathway, phosphorylating Akt and inhibiting the pro-apoptotic factors Bad and GSK-3β, while also stimulating mTOR-dependent processes that support protein synthesis, cell growth, and metabolism (Kowalska et al., 2026).

GLP-1 RAs are not uniformly CNS-penetrant, and this heterogeneity has direct implications for interpreting comparative efficacy data. Preclinical and imaging-based evidence indicates that CNS penetration differs meaningfully across agents, informing the receptor-occupancy assumptions that underlie dose selection in ongoing PD and AD trials (West et al., 2025).

GLP-1R is also expressed on vagal afferent neurons innervating the stomach and hepatic portal vein, providing a route through which peripherally released GLP-1 can influence central circuits without itself crossing the blood–brain barrier (Brierley and de Lartigue, 2022). Centrally, GLP-1 is synthesized locally by preproglucagon neurons in the caudal nucleus tractus solitarii, which project widely to the hypothalamus and to other forebrain, hindbrain, and mesolimbic targets (Trapp and Skoug, 2025). Experimental work manipulating each system independently indicates that peripheral and central GLP-1 signaling act through largely separate circuits that suppress feeding additively, rather than through a single unified gut-to-brain pathway (Brierley et al., 2021). The co-localisation of GLP-1R with dopaminergic neurons in the substantia nigra provides a direct anatomical basis for the mechanistic hypotheses explored in Section 4 and the PD-specific trial evidence reviewed in Section 5 (Reich and Hölscher, 2022).

The principal intracellular cascades downstream of GLP-1R activation, culminating in synaptic and anti-apoptotic effects, are summarized schematically in Figure 1.

**FIGURE 1:**
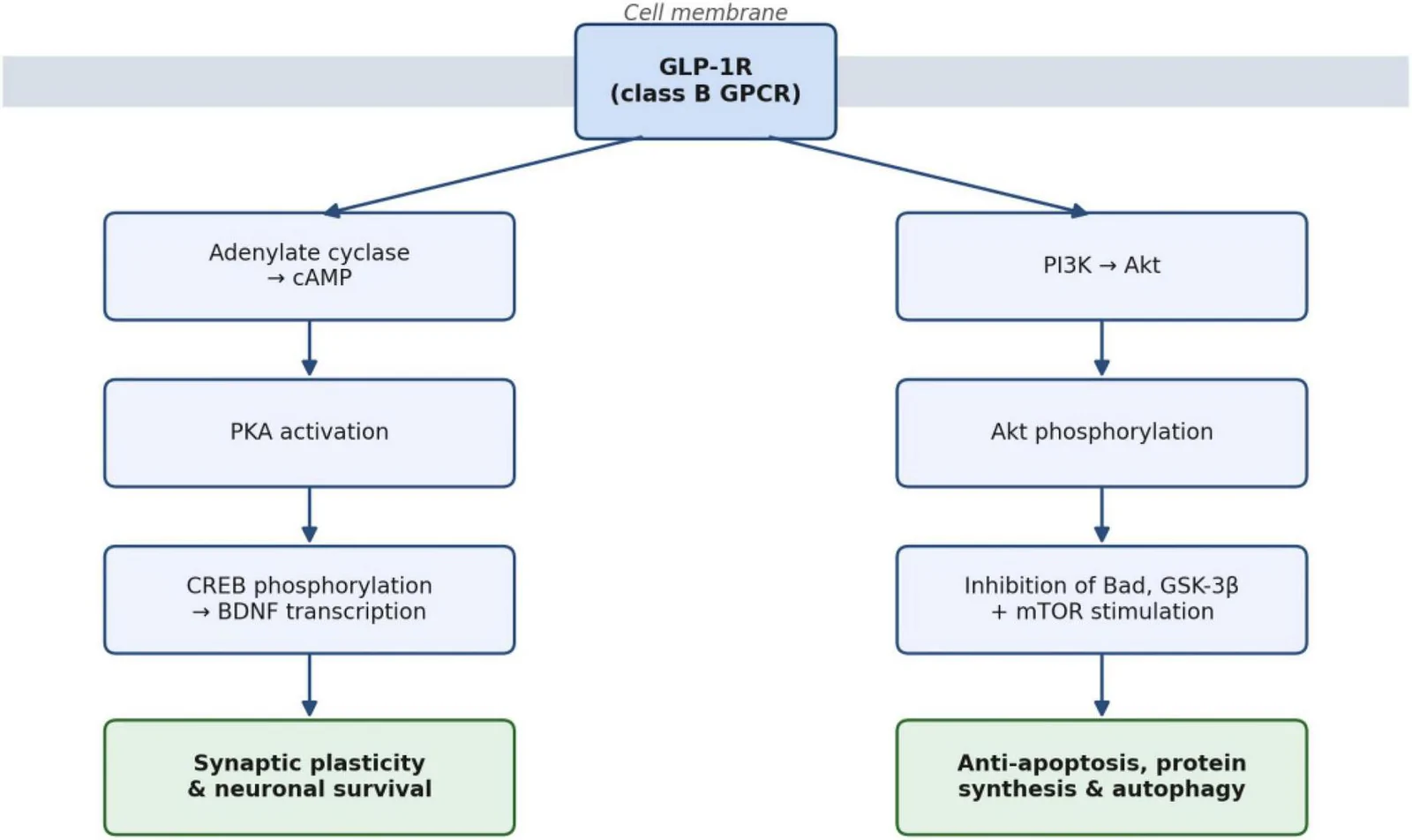
Principal intracellular signaling cascades downstream of GLP-1 receptor activation. GLP-1R engages both the adenylate cyclase/PKA/CREB/BDNF axis, supporting synaptic plasticity and neuronal survival, and the PI3K/Akt axis, which inhibits the pro-apoptotic mediators Bad and GSK-3β and stimulates mTOR-dependent protein synthesis and autophagy regulation (Kowalska et al., 2026).

## 4 Neuroprotective mechanisms of GLP-1 receptor agonism

### 4.1 Anti-neuroinflammatory effects

Neuroinflammation is increasingly recognized as a central contributor to the pathogenesis of neurodegenerative diseases, including PD and AD. Persistent accumulation of misfolded proteins activates microglia, the resident immune cells of the central nervous system, driving overproduction of pro-inflammatory cytokines such as IL-6, IL-1β, and TNF-α; sustained activation of these pathways contributes to neuronal dysfunction, oxidative stress, and progressive neurodegeneration (Shi and Yong, 2025).

Experimental evidence indicates that GLP-1 RAs attenuate this response by suppressing microglial activation and promoting a phenotypic shift from the pro-inflammatory M1 state toward the anti-inflammatory M2 state, in part through inhibition of the NF-κB signaling pathway. This microglial repolarisation is paralleled by a reduction in astrocyte reactivity (Gao et al., 2023; Wang et al., 2024). Clinically, this anti-inflammatory mechanism is one of the more direct candidate explanations for the reductions in fluid inflammatory biomarkers (e.g., plasma C-reactive protein) observed in the EVOKE/EVOKE+ programme despite the absence of cognitive benefit (Section 6); the dissociation between biomarker and clinical response is discussed further in Section “8 Biomarkers of GLP-1 RA neuroprotective response.”

### 4.2 Mitochondrial protection and biogenesis

Mitochondrial dysfunction is a hallmark of neurodegenerative disease, characterized by impaired ATP production, increased oxidative stress, and progressive neuronal injury (Spezani and Mandarim-de-Lacerda, 2026). Experimental studies indicate that GLP-1 RAs preserve mitochondrial function by enhancing mitochondrial biogenesis through upregulation of PGC-1α and SIRT-1 signaling, while improving mitochondrial complex I and II activity and cellular ATP production (Athauda et al., 2026). GLP-1R activation additionally reduces mitochondrial oxidative stress, stabilizes mitochondrial homeostasis, and suppresses apoptosis, thereby increasing neuronal survival in preclinical models of neurodegeneration (Luna-Marco et al., 2023). Because mitochondrial dysfunction is thought to precede overt neuronal loss in both PD and AD, this mechanism is most plausibly relevant to early-stage or prodromal intervention, consistent with the rationale for earlier patient enrolment discussed in Section “10 Future directions.”

### 4.3 Autophagy, proteostasis, and anti-apoptotic signaling

Autophagy is an essential cellular process responsible for degrading damaged organelles and misfolded proteins, thereby maintaining neuronal homeostasis (Nixon and Rubinsztein, 2024). Failure of autophagy contributes to the accumulation of pathological protein aggregates, including α-synuclein in PD and amyloid-β in AD (Nixon, 2024). Experimental evidence indicates that GLP-1 RAs enhance autophagic activity by regulating the mTOR signaling pathway, promoting degradation and clearance of toxic protein deposits and reducing protein-induced cellular stress (Maiese, 2026; Sioufi et al., 2026). GLP-1R activation may also support proteostasis where the ubiquitin–proteasome system is failing. In a 6-hydroxydopamine (6-OHDA) rat model of PD, in which the neurotoxin 6-OHDA selectively destroys dopaminergic neurons to reproduce a Parkinsonian phenotype, ubiquitin–proteasome and autophagy dysfunction are known contributors to cell death; treatment with the GLP-1 RAs liraglutide or semaglutide reversed the resulting decline in cell viability (Meca et al., 2024).

Autophagy and apoptosis are mechanistically intertwined, sharing downstream effectors including PI3K/Akt and mTOR signaling, and are considered jointly here for that reason. GLP-1R activation protects neurons from apoptosis across several toxin-based experimental models. In a rotenone-induced model of PD, pretreatment with a protease-resistant GLP-1 analogue restored expression of the anti-apoptotic protein Bcl-2 and suppressed activation of caspase-3, which rotenone would otherwise continue to trigger (Li et al., 2016). Similarly, in human neuroblastoma cells challenged with hydrogen peroxide or 6-OHDA, GLP-1R stimulation shifted the balance of apoptotic regulators, suppressing the pro-apoptotic protein Bax while raising Bcl-2, and lowered caspase-3 activity; this effect was abolished by inhibiting the PI3K pathway downstream of the receptor (Li et al., 2010). Studies using GLP-1R knockout mice demonstrated that this cytoprotective effect is receptor-dependent, since treatment failed to protect cells when the receptor was absent (Li et al., 2009). Clinically, this convergent autophagy/anti-apoptotic mechanism offers one candidate biological explanation for the persistence of motor benefit after drug washout observed in the Exenatide-PD and LIXIPARK trials (Section 5): a purely symptomatic dopaminergic mechanism would not be expected to outlast drug clearance, whereas preserved neuronal viability would.

### 4.4 Synaptic plasticity and neurogenesis

In an APP/PS1 mouse model of AD, long-term treatment with liraglutide preserved hippocampal synaptic connectivity, prevented synapse loss, and improved performance on memory and object-recognition tasks relative to untreated controls (McClean et al., 2011). In a fast-aging AD mouse model, liraglutide similarly improved memory performance and increased hippocampal CA1 neuronal numbers (Hansen et al., 2015). Together, these findings suggest that GLP-1 RAs can preserve existing synaptic architecture and may support hippocampal neurogenesis during cognitive decline. Clinically, preserved synaptic connectivity would be expected to manifest as slowed decline rather than as improvement in already-established deficits; this is broadly consistent with the modest, imaging-restricted signal observed in the ELAD trial (Section 6) rather than a categorical cognitive benefit, and underscores why earlier intervention, before substantial synapse loss has occurred, may be mechanistically important (Section 10).

### 4.5 Cerebrovascular effects

GLP-1R activation also exerts direct effects on cerebral vasculature. In rats, GLP-1R stimulation dilated cerebral arterioles and increased cerebral blood flow, conferring protection against ischaemic stroke (Nizari et al., 2021). In the APP/PS1 mouse model of AD, liraglutide treatment reduced the number of cerebral microaneurysms and prevented vascular leakage (Kelly et al., 2015), a finding consistent with the vascular fragility described in models of amyloid angiopathy (Christie et al., 2001) and with evidence that GLP-1R signaling restores aquaporin-4 polarization in reactive astrocytes to promote amyloid-β clearance (Sasaki et al., 2024). This vascular mechanism is mechanistically distinct from direct neuronal GLP-1R engagement and may plausibly contribute to the epidemiological association between GLP-1 RA use and lower dementia incidence described in Section 1, independent of any effect on amyloid or tau pathology specifically; this indirect, cardiovascular-mediated pathway is discussed further in Section “9 Challenges, limitations, and unresolved questions.”

### 4.6 Glymphatic system enhancement

The glymphatic system, which clears interstitial metabolic waste, including amyloid-β, via perivascular aquaporin-4-dependent flow, is impaired in both PD and AD. Restoration of astrocytic aquaporin-4 polarization by GLP-1R signaling (Sasaki et al., 2024) suggests a mechanistic link between GLP-1 RA treatment and improved glymphatic clearance, complementing the receptor’s direct anti-inflammatory and vascular effects and providing a further route by which sustained GLP-1R engagement could plausibly modify disease trajectory, though direct clinical evidence for this pathway in PD or AD remains limited.

The mechanistic domains reviewed in this section are summarized together in Figure 2, which maps each pathway to its supporting evidence and to the principal preclinical model systems in which it has been demonstrated. Figure 2 depicts autophagy/proteostasis and anti-apoptotic signaling as two adjacent boxes, reflecting their distinct assay readouts in the underlying preclinical literature, even though Section 4.3 above discusses them jointly given their shared upstream signaling.

**FIGURE 2:**
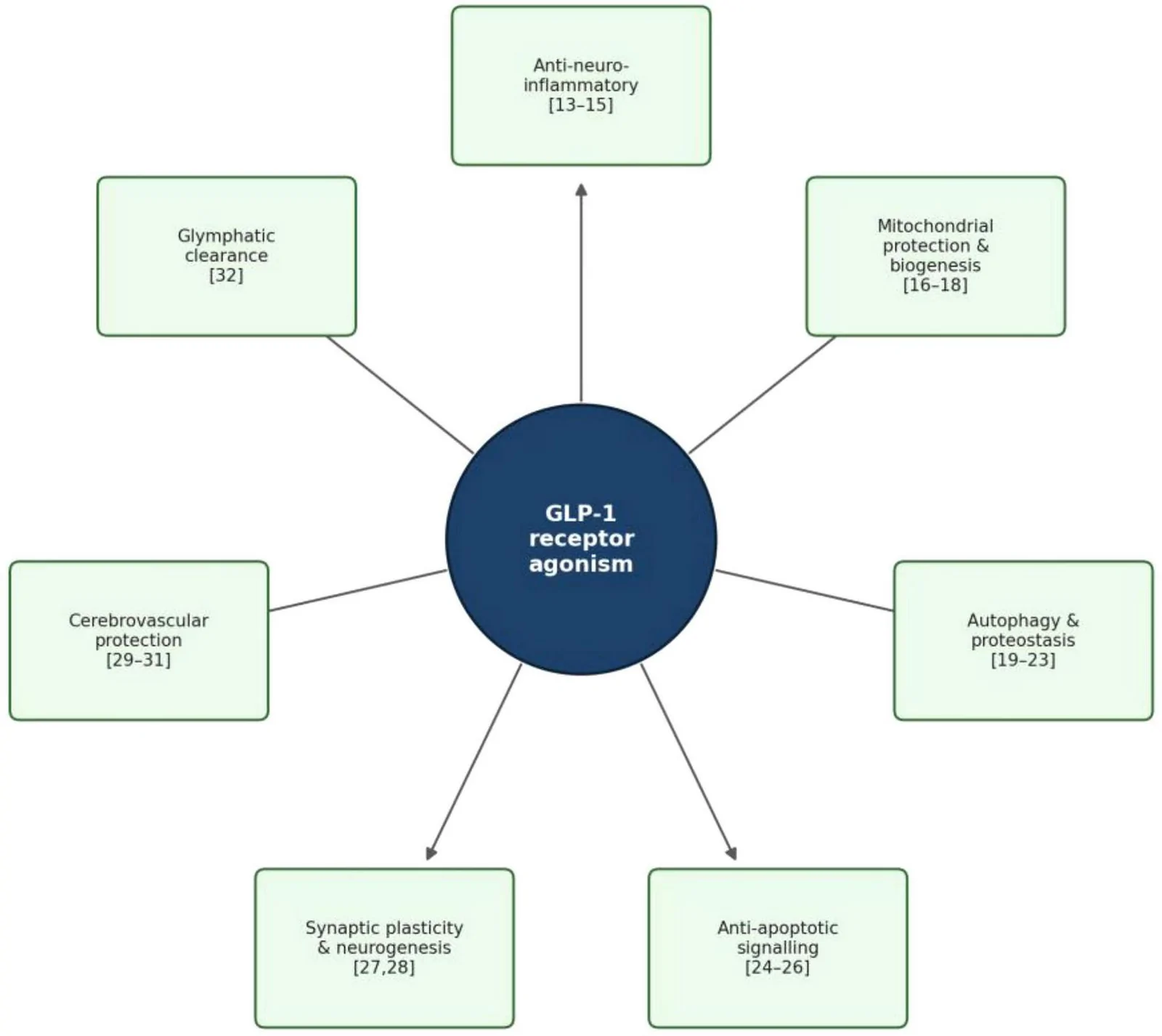
Overview of the principal neuroprotective mechanism domains attributed to GLP-1 receptor agonism in preclinical models of Parkinson’s and Alzheimer’s disease, evaluated primarily in toxin-based rodent and cell-culture models (6-OHDA- and rotenone-induced PD models; hydrogen-peroxide-challenged neuroblastoma cells) and in transgenic AD models (APP/PS1, senescence-accelerated mice), with supporting references for each domain as shown.

## 5 GLP-1 receptor agonists in Parkinson’s disease: clinical evidence

The randomized controlled trials discussed in this section and in Section 6 are summarized in Table 1, which juxtaposes trial phase, population, registry identifier, design, and primary outcome – including effect sizes, confidence intervals, and *p*-values where reported – to facilitate direct comparison across the PD and AD programmes.

**TABLE 1:** Summary of major randomized controlled trials of GLP-1 receptor agonists in Parkinson’s and Alzheimer’s disease.

Trial (agent)	Phase/disease	*N*	Registry ID	Design and duration	Primary outcome and key result	References
Exenatide-PD (exenatide)	II/PD	60 (30/30)	NCT01971242	RCT; 48-week treatment + 12-week washout	MDS-UPDRS III (off-med, week 60): benefit persisted after washout (∼3.5-pt between-group difference)	Athauda et al., 2017
Exenatide-PD3 (exenatide)	III / PD	194 (97/97)	NCT04232969	RCT; 96-week treatment; extended-release weekly formulation	MDS-UPDRS III (off-med): no significant difference (adj. coefficient 0.92; 95% CI −1.56 to 3.39; *p* = 0.47); SAEs 9% vs. 11%	Vijiaratnam et al., 2025
LIXIPARK (lixisenatide)	II/PD	156 (78/78)	NCT03439943	RCT; 12-month treatment (20 μg SC daily) + 2-month washout	MDS-UPDRS III: +3.08-pt between-group difference (95% CI 0.86–5.30; *p* = 0.007); persisted post-washout; GI AEs common (nausea 46%, vomiting 13%)	Meissner et al., 2024
MOST-ABLE (oral semaglutide)	II / PD	99 (∼33/33/33; 1:1:1)	jRCT2051230090	RCT; 36–48-week treatment (7 or 14 mg) + 12-week washout	MDS-UPDRS III: ongoing (dose-finding study; not powered as a definitive efficacy trial)	Kimura et al., 2025
ELAD (liraglutide)	IIb/AD	204	NCT01843075	RCT; 12-month treatment (1.8 mg SC daily)	Primary (cerebral glucose metabolic rate, FDG-PET): no significant difference (*p* = 0.14); secondary ADAS-Exec improved (*p* = 0.01); ADCS-ADL, CDR-SB unchanged	Edison et al., 2026; Femminella et al., 2019
EVOKE / EVOKE+ (oral semaglutide)	III / AD	3,808 (1855/1953)	NCT04777396 / NCT04777409	RCT; up to 156-week treatment (14 mg flexible dose)	CDR-SB at week 104: EVOKE diff −0.08 (95% CI −0.35 to 0.20; *p* = 0.57); EVOKE+ diff 0.10 (95% CI −0.17 to 0.38; *p* = 0.46); neither met primary endpoint	Cummings et al., 2022, 2025, 2026
Exenatide pilot (exenatide)	II / AD-MCI	Small pilot cohort	–	RCT; 18-month treatment	Safety and biomarker signal only; no cognitive benefit; limited EV Aβ42 signal; underpowered after early termination	Mullins et al., 2019

SAE, serious adverse event; AE, adverse event; GI, gastrointestinal; EV, extracellular vesicle.

### 5.1 Exenatide in Parkinson’s disease: the exenatide-PD trial

#### 5.1.1 Trial design

The first substantive clinical evidence for GLP-1 RAs in PD came from a phase II, double-blind, placebo-controlled trial conducted by Athauda and colleagues (ClinicalTrials.gov NCT01971242). Sixty patients with moderate PD were randomized evenly to weekly 2 mg subcutaneous exenatide or placebo for 48 weeks, followed by a 12-week washout period before final evaluation. The washout design was intended to distinguish a disease-modifying effect from symptomatic benefit: persistence of clinical improvement after drug clearance would be inconsistent with a purely symptomatic mechanism (Athauda et al., 2017).

#### 5.1.2 Primary outcome

The primary outcome was the change in MDS-UPDRS Part III motor score at 60 weeks, assessed in the practically defined “off-medication” state after washout. Patients receiving exenatide improved by a mean of 2.5 points while those receiving placebo worsened by a mean of 1 point, a between-group difference of approximately 3.5 points. Because this motor benefit persisted after treatment discontinuation, the authors interpreted the finding as consistent with a disease-modifying rather than purely symptomatic effect (Athauda et al., 2017).

#### 5.1.3 Biomarker sub-study

A smaller biomarker sub-study examined cerebrospinal fluid markers of insulin signaling alongside plasma neurofilament light chain (NfL) and α-synuclein to probe underlying cellular mechanisms. A secondary analysis using neuronal-derived extracellular vesicles additionally found that exenatide engaged Akt/mTOR signaling in parallel with the observed clinical motor benefit, offering indirect evidence of brain insulin sensitisation as a candidate mechanism linking peripheral drug exposure to central biological effect (Athauda et al., 2019). These exploratory analyses were nonetheless underpowered given the small sample size and yielded mixed, inconclusive results overall, as acknowledged by the trial authors (Athauda et al., 2017).

### 5.2 Exenatide-PD3 trial

Building on the phase II findings, a larger UK-wide phase III trial, Exenatide-PD3 (ClinicalTrials.gov NCT04232969), enrolled 194 patients with mild-to-moderate PD (97 randomized to exenatide, 97 to placebo), randomized to weekly 2 mg extended-release exenatide or placebo for 96 weeks, again using the off-medication MDS-UPDRS Part III score as the primary outcome. At 96 weeks, off-medication scores had worsened by a mean of 5.7 points in the exenatide group and 4.5 points in the placebo group; the trial found no significant difference in motor progression between groups (adjusted coefficient for the effect of exenatide 0.92, 95% CI −1.56 to 3.39; *p* = 0.47), with similar serious adverse event rates in both arms (9% exenatide vs. 11% placebo) (Vijiaratnam et al., 2025). This null result is an important corrective for the field: in contrast to the positive phase II Exenatide-PD and LIXIPARK signals described here, it indicates that the phase II benefit did not replicate at larger scale, longer duration, and with an extended-release rather than immediate-release formulation (Section 7). The wide confidence interval around the point estimate also reflects limited statistical power to exclude a modest true effect or to support definitive subgroup analyses (Section 9), and the result tempers the optimism generated by the preclinical and phase II biomarker data discussed in Sections 4 and 5.1.

### 5.3 Lixisenatide in Parkinson’s disease: the LIXIPARK trial

The subsequent major dataset came from the phase II LIXIPARK trial (ClinicalTrials.gov NCT03439943), which randomized 156 patients with early PD (78 per group) to once-daily subcutaneous lixisenatide (20 μg, with a pre-specified reduction to 10 μg permitted for participants with tolerability issues) or placebo for 12 months, followed by a 2-month washout. In contrast to the null Exenatide-PD3 result, LIXIPARK reported a positive primary outcome: at 12 months, on-medication MDS-UPDRS Part III scores changed by −0.04 points (improvement) with lixisenatide and worsened by 3.04 points with placebo, a between-group difference of 3.08 points (95% CI 0.86–5.30; *p* = 0.007) favoring lixisenatide. This benefit persisted after the 2-month washout (off-medication scores 17.7 vs. 20.6 with placebo), and because it persisted after treatment discontinuation, the authors interpreted it as consistent with disease modification. Gastrointestinal adverse effects were common (nausea in 46% and vomiting in 13% of the lixisenatide group), consistent with the known tolerability profile of this drug class, and lixisenatide did not improve non-motor symptoms or other secondary endpoints (Meissner et al., 2024).

### 5.4 Semaglutide in Parkinson’s disease

Given the divergent results with injectable agents, attention has turned to oral formulations. The phase II MOST-ABLE trial (Japan Registry of Clinical Trials jRCT2051230090) is currently ongoing and is evaluating once-daily oral semaglutide (7 mg or 14 mg, following a 4-week titration from 3 mg) against placebo, added to standard therapy, in 99 patients with early-stage PD (Hoehn and Yahr stages 1–2.5), randomized 1:1:1 across the two active-dose arms and placebo (approximately 33 participants per arm). As in the lixisenatide trials, the primary outcome is change in off-medication MDS-UPDRS Part III motor score after 36–48 weeks of treatment and a 12-week washout. Because the trial is a three-arm phase II dose-finding study rather than a two-arm confirmatory trial, its per-arm sample size is smaller than that of a definitive phase III trial and the study is not intended to provide the same statistical power for detecting a modest treatment effect; results should accordingly be interpreted as hypothesis-generating for dose selection and safety rather than as definitive evidence of efficacy. This trial is nonetheless of particular interest given the pronounced anti-inflammatory signal associated with oral semaglutide, positioning it as a candidate test of whether disease-modifying benefit can be achieved with a non-injectable formulation (Kimura et al., 2025).

### 5.5 Observational and registry data

Beyond controlled trials, large-scale real-world data provide complementary evidence on the association between GLP-1 RA use and PD risk. Population-based studies using electronic health records from the United Kingdom and Denmark, following thousands of patients with diabetes over several years, consistently report a lower incidence of PD diagnosis among GLP-1 RA users compared with users of other antidiabetic drug classes (Brauer et al., 2020). Although observational designs cannot establish causation, the consistency of this signal across independent European registries strengthens the case for a genuine, if modest, protective association (Engström et al., 2026; Gamborg et al., 2025). A specific limitation of this literature deserves emphasis: because all participants in these registry studies were already being treated for T2DM, the observed association cannot readily distinguish a direct neuroprotective effect of GLP-1 RAs from an indirect benefit of improved glycaemic control, weight loss, or reduced vascular disease burden relative to comparator antidiabetic drug classes; this ambiguity is revisited in Section 9 and mirrors the interpretive challenge that affects the interventional trial literature more broadly.

### 5.6 Non-motor PD benefits

Beyond motor outcomes, clinical evidence suggests that GLP-1 RAs may benefit non-motor symptoms of PD. *Post-hoc* analysis of the Exenatide-PD trial data showed significant improvements in mood and psychological wellbeing among patients receiving weekly exenatide, particularly for depressive and apathetic symptoms (Athauda et al., 2018). Longer-term observational data further suggest benefits for autonomic function, including weight stability and gastrointestinal function, and secondary outcomes have suggested a possible cognitive benefit. Because non-motor symptoms often contribute more to quality of life than motor impairment itself, these secondary findings represent a clinically meaningful, if still preliminary, rationale for continued investigation (Athauda et al., 2018).

### 5.7 Dopamine transporter (DAT-SPECT) as a neuroprotection endpoint

To distinguish genuine neuroprotection from symptomatic masking, recent trials have incorporated dopamine transporter single-photon emission computed tomography (DAT-SPECT), which allows *in vivo* quantification of surviving dopaminergic nerve terminals. In early studies, patients receiving GLP-1 RAs showed a slower decline in dopamine transporter availability than placebo-treated patients, consistent with preservation of dopaminergic terminals. However, the regulatory qualification of DAT-SPECT as a primary endpoint for disease modification remains contested, since factors such as transient drug-related changes in transporter regulation can alter imaging signal independently of true neuronal survival; regulatory bodies currently regard DAT-SPECT as a supportive secondary measure rather than definitive proof of disease modification (Akdemir et al., 2021).

The chronological relationship between preclinical discovery and the initiation and reporting of the phase II–III trials discussed in this section and in Section 6 is summarized in Figure 3.

**FIGURE 3:**
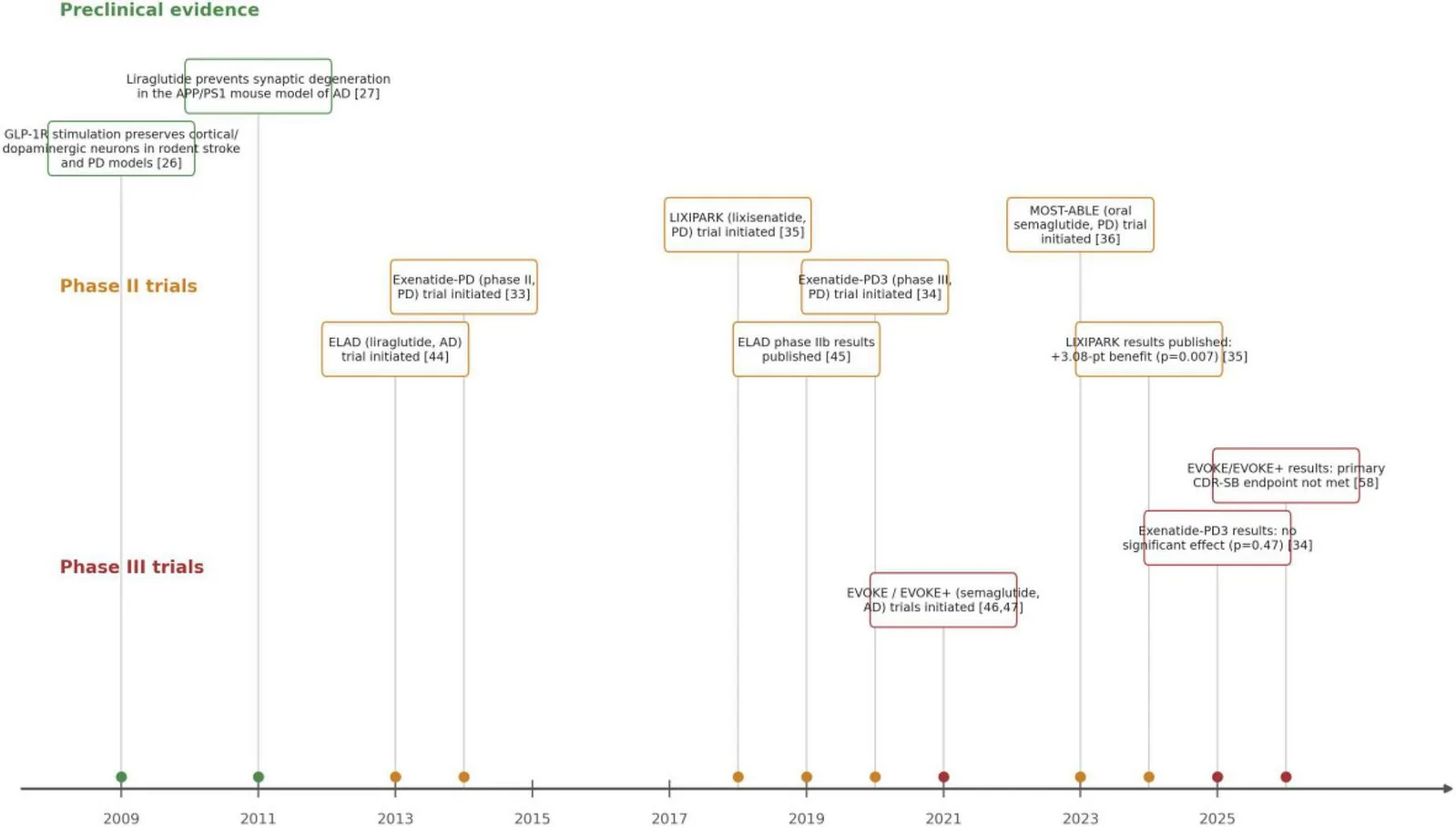
Temporal evolution of GLP-1 receptor agonist research in Parkinson’s and Alzheimer’s disease, from preclinical proof-of-concept through phase II and phase III clinical trials. Selected trial initiation and results-reporting milestones are shown; reference numbers correspond to the citation list.

## 6 GLP-1 receptor agonists in Alzheimer’s disease: clinical evidence

Clinical translation of GLP-1 RAs as disease-modifying therapies in AD has been more consistently disappointing than in PD, with every completed randomized trial to date failing to meet its primary clinical or imaging endpoint, despite biomarker engagement signals in several programmes. Mechanistic plausibility remains strong: GLP-1R agonism intersects with brain energy metabolism, neuroinflammation, and insulin signaling, processes implicated in AD pathophysiology (Koychev et al., 2024), yet despite robust peripheral metabolic effects, the clinical literature has not yet demonstrated a corresponding slowing of established AD progression (Yang and Liang, 2026).

### 6.1 Liraglutide: the ELAD trial

Liraglutide provided the earliest phase II test of this hypothesis. The ELAD trial (ClinicalTrials.gov NCT01843075), a 12-month, multicentre, double-blind, phase IIb randomized trial of liraglutide 1.8 mg daily versus placebo in 204 participants with mild AD, assessed cerebral glucose metabolism using fluorodeoxyglucose positron emission tomography (FDG-PET), MRI, and psychometric evaluation as pre-specified outcomes (Femminella et al., 2019). Contrary to an earlier, less precise characterisation of this trial in prior drafts of this review, ELAD did not achieve a statistically significant effect on its pre-specified primary imaging outcome: cerebral glucose metabolic rate did not differ significantly between groups (difference −0.17; 95% CI −0.39 to 0.06; *p* = 0.14). Among secondary outcomes, the ADAS-Exec cognitive subscore was modestly better with liraglutide (difference 0.15; 95% CI 0.03–0.28; *p* = 0.01), but no significant differences were observed for the ADCS-ADL functional scale (difference −0.58; 95% CI −3.13 to 1.97; *p* = 0.65) or the CDR-SB global measure (difference −0.06; 95% CI −0.57 to 0.44; *p* = 0.81). Liraglutide was generally safe and well tolerated in this non-diabetic AD population (Edison et al., 2026). Taken as a whole, ELAD therefore provides, at most, a modest and imaging-restricted signal of biological engagement rather than evidence of a primary-endpoint benefit, a distinction that is important for accurately weighing this trial against the subsequent phase III programme.

### 6.2 Semaglutide: the EVOKE and EVOKE+ trials

Semaglutide is now the most advanced GLP-1 RA candidate in large-scale AD disease-modification testing. EVOKE (ClinicalTrials.gov NCT04777396) and EVOKE+ (NCT04777409) are formally two separate, independently powered phase III trials, both evaluating oral semaglutide 14 mg (flexible dose) against placebo in participants with amyloid-confirmed, early symptomatic AD (mild cognitive impairment or mild dementia), using change in CDR-SB from baseline to week 104 as the primary endpoint over a planned treatment duration of up to 156 weeks (Cummings et al., 2022, 2025). The two trials differ in eligibility: EVOKE+ additionally enrolled a small proportion of participants with cerebral small-vessel pathology (54 of 1,953 participants, 2.8%), permitting limited assessment of vascular co-pathology, whereas EVOKE excluded this group.

Neither trial met its primary endpoint. In EVOKE (928 semaglutide, 927 placebo), mean CDR-SB change from baseline to week 104 was 2.3 points with semaglutide versus 2.3 points with placebo (estimated difference −0.08; 95% CI −0.35 to 0.20; *p* = 0.57). In EVOKE+ (976 semaglutide, 977 placebo), mean change was 2.2 versus 2.1 points (estimated difference 0.10; 95% CI −0.17 to 0.38; *p* = 0.46) (Cummings et al., 2026). Secondary cognitive and functional endpoints (ADCS-ADL-MCI, MMSE, ADAS-Cog) showed the same neutral pattern in both trials, and a pooled analysis found no delay in progression from MCI to mild AD with semaglutide.

A pre-specified cerebrospinal fluid sub-study in 199 participants across the pooled programme nonetheless demonstrated a 10% reduction relative to placebo in p-tau181, p-tau217, total tau, YKL-40, neurogranin, and soluble TREM2 (sTREM2), alongside a significant reduction in plasma high-sensitivity CRP and an increase in plasma glial fibrillary acidic protein (GFAP), with plasma neurofilament light chain (NfL) unchanged (Cummings et al., 2026). This biomarker effect size was smaller than that reported for anti-amyloid monoclonal antibodies. The trial investigators proposed insufficient CNS bioavailability of oral semaglutide as one possible explanation; however, this attribution should be treated as a hypothesis rather than an established conclusion, since the trial was not designed to formally test it, and the measurable reductions in CSF tau and neuroinflammatory markers argue against a simple, complete failure of target engagement. A mechanistically important, currently unresolved question is therefore whether the null clinical outcome instead reflects adequate central biological engagement that was nonetheless insufficient, at the disease stage enrolled, to alter the already-established trajectory of amyloid and tau pathology – in which case treatment timing relative to the decades-long biology of AD, rather than drug exposure per se, may be the more important limiting factor (Section 9).

### 6.3 Exenatide in Alzheimer’s disease

Evidence for exenatide in AD is comparatively limited. A *post-hoc* analysis of the EXSCEL cardiovascular outcomes trial found that once-weekly exenatide modulated inflammatory mediator proteins in AD-relevant pathways, an effect concentrated among patients over 65 years of age or with prior cardiovascular disease (Koychev et al., 2024). In a separate, National Institute on Aging-sponsored phase II pilot trial in early AD and mild cognitive impairment, using biomarker-enriched enrolment, exenatide had an acceptable safety and expected metabolic profile but did not detect improvement in clinical or cognitive outcomes over 18 months. CSF Aβ42/tau/p-tau were measured alongside neuronal-derived plasma extracellular vesicle (EV) Aβ42 and insulin receptor substrate-1 (IRS-1) phosphorylation as a marker of insulin resistance; the trial found a reduction in EV Aβ42 without a corresponding change in the other markers. Biomarker findings were therefore exploratory and limited to this single EV-based readout rather than classic CSF or plasma AD biomarkers or cognitive endpoints, and the study was underpowered following early termination (Mullins et al., 2019). While such EV-based signals are of interest as potential markers of target engagement, they have not been shown to translate into cognitive benefit.

Collectively, the AD trial literature describes a consistent disconnect between systemic metabolic improvement and neurodegenerative progression: incretin therapy reliably improves glucose tolerance and cardiometabolic parameters and is associated with lower dementia incidence in observational data, yet has repeatedly produced neutral primary outcomes in interventional AD trials (Santos et al., 2026). GLP-1R agonism may be most effective in the subset of patients in whom brain insulin-signaling dysfunction is the dominant driver of pathology; accordingly, stratification variables such as insulin resistance, circulating or CSF insulin measures, and FDG-PET are increasingly proposed to identify GLP-1 RA-responsive subgroups, while benefit may be attenuated in patients whose disease trajectory is predominantly amyloid- or tau-driven rather than insulin-linked (Dahshan et al., 2026; Fallot et al., 2026). Beyond these indirect metabolic and inflammatory pathways, a direct biophysical interaction between GLP-1 RAs and pathogenic protein species has also been proposed: *in vitro*, GLP-1 RAs bind directly to amyloid-β monomers and inhibit the nucleation step of toxic amyloid-β42 aggregation, a mechanism distinct from, and potentially complementary to, the receptor-mediated anti-inflammatory and pro-autophagic effects described in Section “4 Neuroprotective mechanisms of GLP-1 receptor agonism” (Fallot et al., 2026). At present, there is no evidence that GLP-1 RAs alter the course of established AD. Combination strategies pairing GLP-1 RAs with anti-amyloid antibodies such as lecanemab have been proposed to unite the metabolic, anti-inflammatory, and neurovascular effects of incretin therapy with direct action on amyloid pathology (Yang and Liang, 2026).

### 6.4 Comparison of PD versus AD trial outcomes

Direct comparison of the PD and AD trial programmes highlights a disease-specific pattern rather than a uniform class effect. In PD, two of three completed randomized trials (Exenatide-PD and LIXIPARK) reported statistically significant, clinically modest benefit on the primary motor endpoint that persisted after washout, with only the largest and longest trial (Exenatide-PD3, using an extended-release weekly formulation) returning a null result. In AD, by contrast, every completed randomized trial to date, spanning phase IIb (ELAD) through phase III (EVOKE/EVOKE+), has failed to demonstrate benefit on its primary clinical or imaging endpoint, despite biomarker evidence of target engagement in several programmes.

Several factors may plausibly explain this divergence. First, the PD trials have used a mechanistically motivated washout design specifically intended to unmask disease modification, whereas the AD trials have relied on continuous longitudinal change in a composite functional scale (CDR-SB) that may be less sensitive to small biological effects over the 104-week assessment window. Second, PD outcome measures (MDS-UPDRS Part III) capture a relatively circumscribed dopaminergic motor circuit with a well-characterized pathophysiology, whereas AD progression reflects a more diffuse, multi-network pathological process spanning amyloid, tau, neuroinflammatory, and vascular components, any one of which may dominate in a given patient. Third, the PD trial populations may be enriched for a subset of patients whose disease is more proximally linked to insulin signaling and nigral dopaminergic neurodegeneration, whereas amyloid- and tau-driven AD pathology may be comparatively insulin-independent in a larger proportion of patients (Section 6.3). These differences argue against extrapolating a positive or negative signal from one disease to the other, and support disease-specific, rather than pan-neurodegenerative, trial design going forward (Section 10).

## 7 Comparative pharmacology: exenatide vs. liraglutide vs. semaglutide vs. tirzepatide

Although exenatide, liraglutide, semaglutide, and tirzepatide are all incretin-based agents, they differ meaningfully in receptor pharmacology, molecular structure, bioavailability, and brain-penetration properties, with direct consequences for their neuroprotective potential. These differences are summarized in Table 2 and elaborated below. Exenatide and liraglutide are selective GLP-1 receptor agonists, whereas tirzepatide is a unimolecular co-agonist of the GLP-1 and GIP receptors. Pharmacological characterisation shows that tirzepatide is an imbalanced agonist: it binds the GIP receptor with affinity and potency approximately equal to native GIP, but binds the GLP-1 receptor with roughly five-fold lower affinity and substantially lower potency (approximately 18- to 20-fold lower in cAMP signaling assays) than native GLP-1, so that its overall pharmacological activity is weighted more heavily toward GIP-receptor engagement than toward classical GLP-1-receptor signaling (Willard et al., 2020). This imbalance has been proposed to confer an additive neurotrophic and anti-inflammatory effect beyond GLP-1 mono-agonism alone (Alshehri et al., 2025; Rhea et al., 2024), but it also means that tirzepatide’s neuroprotective potential cannot be assumed to scale directly with its nominal classification as a GLP-1 RA, and mechanistic data specific to its CNS effects remain considerably more limited than for the selective GLP-1 RAs.

**TABLE 2:** Comparative pharmacology of GLP-1 receptor agonists relevant to central nervous system engagement.

Agent	Receptor target and CNS penetration	Bioavailability	Administration	Key CNS/safety consideration	References
Exenatide	GLP-1R (selective); small peptide, measurable CSF detectability (immediate-release)	∼65%–75% SC (IR); ∼25% SC (ER, relative to IR)	Weekly (ER) or twice-daily (IR), subcutaneous	IR CSF penetration and motor benefit not replicated with ER weekly formulation	Athauda et al., 2017; Hunter and Hölscher, 2012; Vijiaratnam et al., 2025
Liraglutide	GLP-1R (selective); GLP-1R-dependent uptake into hypothalamic circuits	∼55% SC	Once daily, subcutaneous	Preserves cerebral glucose metabolism on FDG-PET in AD models; no significant primary-endpoint benefit in ELAD	Edison et al., 2026; Femminella et al., 2019; Hunter and Hölscher, 2012
Semaglutide	GLP-1R (selective); circumventricular-organ-mediated access; 94% homology to native GLP-1, extended half-life via albumin binding	∼89% SC; ∼0.4%–1% oral	Once weekly (SC) or once daily (oral)	Largest biomarker dataset (EVOKE/EVOKE+ CSF sub-study) but no clinical benefit to date	Cummings et al., 2026; Hunter and Hölscher, 2012; Meier, 2021
Tirzepatide	GLP-1R/GIP co-agonist, imbalanced toward GIPR (∼5-fold lower GLP-1R affinity than native GLP-1)	Not established in humans; limited published PK data	Once weekly, subcutaneous	Slower BBB crossing (∼6 h post-injection); high albumin binding; CNS neurodegeneration trial data still limited	Alshehri et al., 2025; Rhea et al., 2024; Willard et al., 2020

SC, subcutaneous; IR, immediate-release; ER, extended-release; PK, pharmacokinetic; BBB, blood–brain barrier.

CNS access does not track simply with molecular size or lipophilicity, and available evidence complicates any assumption of a straightforward penetration “hierarchy.” Exenatide, a comparatively small peptide, shows measurable cerebrospinal fluid detectability; liraglutide’s brain-region uptake appears dependent on GLP-1R-mediated transport into hypothalamic circuits; and semaglutide, despite its extended half-life from albumin binding and 94% sequence homology to native GLP-1, appears to act principally through circumventricular organs and adjacent regions rather than broad parenchymal penetration (Hunter and Hölscher, 2012). Tirzepatide crosses the blood–brain barrier more slowly (approximately 6 h post-injection) than albiglutide or dulaglutide, reflecting its high albumin binding (Rhea et al., 2024). These comparative CNS-penetration characteristics are summarized schematically in Figure 4.

**FIGURE 4:**
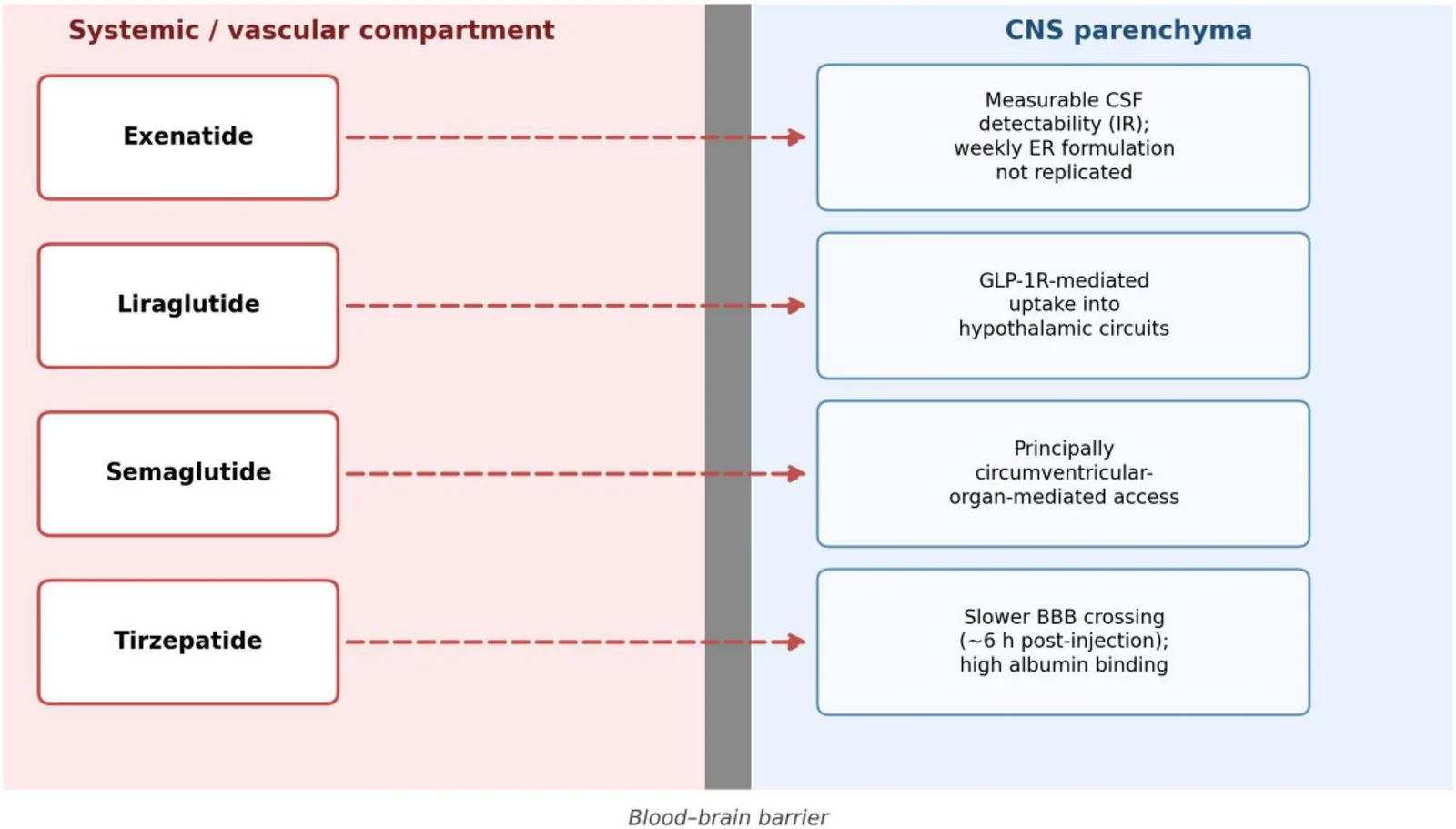
Schematic comparison of central nervous system access across exenatide, liraglutide, semaglutide, and tirzepatide. Dashed arrows indicate that penetration across the blood–brain barrier is partial and agent-specific rather than a simple function of dose; the accompanying notes summarize the principal route or limitation reported for each agent (Hunter and Hölscher, 2012; Rhea et al., 2024).

Taken together, the available CNS-penetration and clinical-trial data do not show a consistent correlation between an agent’s degree of measurable brain exposure and its clinical trial outcome. Lixisenatide, despite modest and incompletely characterized CNS exposure, produced the most convincing motor benefit in PD; immediate-release exenatide showed measurable CSF penetration and phase II benefit, but the same molecule delivered as an extended-release weekly formulation, with presumably similar ultimate brain exposure, was ineffective in the larger, longer Exenatide-PD3 trial; and semaglutide, with the most extensively characterized biomarker engagement of any agent in this class (Section 8), has not yet translated that engagement into clinical benefit in AD. This dissociation suggests that CNS penetration, while probably necessary, is not sufficient for disease modification, and that dosing, treatment duration, formulation (immediate- versus extended-release), and patient selection may be at least as important as absolute brain drug levels in determining trial outcome.

Dosing route further differentiates the class: exenatide, liraglutide, semaglutide, and tirzepatide are all administered subcutaneously (daily for liraglutide; weekly for extended-release exenatide, semaglutide, and tirzepatide), while semaglutide is uniquely available as a once-daily oral tablet. Systemic bioavailability also varies substantially across the class: subcutaneous immediate-release exenatide has an absolute bioavailability of approximately 65–75%, and the extended-release weekly formulation approximately 25% relative to the immediate-release formulation (AstraZeneca Pharmaceuticals LP, 2021); subcutaneous liraglutide has an absolute bioavailability of approximately 55% (Novo Nordisk A/S, 2023); and oral semaglutide has an absolute bioavailability of only approximately 0.4–1% relative to its subcutaneous formulation, reflecting the inherent difficulty of oral peptide delivery (Meier, 2021). Once-weekly subcutaneous regimens plausibly improve adherence in patients with cognitive or motor impairment, but this potential advantage trades off against formulation-specific uncertainty in CNS exposure, illustrated by the negative Exenatide-PD3 result with an extended-release weekly formulation despite prior evidence of CSF penetration and motor benefit with immediate-release exenatide (Athauda et al., 2017; Vijiaratnam et al., 2025).

Safety considerations in neurodegenerative populations warrant particular caution. Gastric motility disorders and dysphagia are common in PD, and the delayed gastric emptying associated with GLP-1 RAs can compound motor fluctuations, reduce oral levodopa absorption, and contribute to frailty-related weight loss, effects that may be amplified by concomitant anticholinergic medication (Trujillo, 2020). This raises a specific interpretive caveat for the positive PD motor trials: if delayed gastric emptying meaningfully altered levodopa pharmacokinetics or absorption in treated participants, this could in principle confound the apparent motor benefit of lixisenatide and exenatide, although the persistence of benefit after washout in both LIXIPARK and Exenatide-PD argues against this being the sole explanation, since any levodopa-absorption effect would be expected to resolve rapidly once the GLP-1 RA is discontinued. A pharmacovigilance study additionally reported an increased reporting frequency of tremor with GLP-1 RA use (adjusted reporting odds ratio 1.78, 95% CI 1.65–1.91); this disproportionality signal warrants cautious interpretation rather than a direct causal inference (Zhao et al., 2024). Taken together, these pharmacological differences justify agent-specific evaluation in future disease-modification trials and argue against extrapolating efficacy or safety findings across the class as a whole.

## 8 Biomarkers of GLP-1 RA neuroprotective response

A recurring limitation across GLP-1 RA trials in neurodegenerative disease is the difficulty of distinguishing pharmacological target engagement from a true disease-modifying effect using clinical endpoints alone, which has motivated increasingly detailed biomarker sub-studies; the principal trial-specific biomarker findings are summarized in their respective trial subsections above (Sections 5.1.3 and 6). This section synthesizes the cross-agent and cross-disease patterns that emerge from those data, and makes explicit a distinction that is easy to elide when reading the trial literature in isolation: a biomarker or target-engagement effect can, and frequently does, occur without corresponding disease modification. A shift in a fluid or imaging biomarker demonstrates that a drug has reached and altered a relevant biological pathway, but does not by itself establish that the pathological trajectory of the disease has been slowed. Keeping this distinction explicit is essential to interpreting the trial literature, and is the central reason biomarker sub-studies cannot substitute for clinical or functional primary endpoints, however encouraging their direction of effect.

Across the EVOKE/EVOKE+, Exenatide-PD, and exenatide-AD programmes, a consistent pattern emerges: pharmacodynamic and neuroinflammatory biomarkers shift in the biologically expected direction more often, and more reliably, than clinical or cognitive endpoints improve. This dissociation was most pronounced in the EVOKE/EVOKE+ CSF sub-study, where measurable reductions in several tau and neuroinflammatory markers accompanied a null clinical result (Section 6.2). Liraglutide has separately been shown to preserve cerebral glucose metabolism on FDG-PET and to correlate with structural outcomes on MRI in an earlier, smaller trial, even though ELAD itself did not reach nominal statistical significance on its cerebral glucose metabolism primary endpoint (Section 6.1) (Edison et al., 2026; Femminella et al., 2019; Gejl et al., 2016).

Collectively, these biomarker data suggest that stratifying patients by baseline insulin resistance and peripheral inflammatory burden may better identify GLP-1 RA-responsive subgroups than unselected enrolment, a hypothesis supported by the *post-hoc* finding that earlier-stage, less insulin-resistant PD patients derived greater benefit from exenatide (Athauda et al., 2017). Future trials will require harmonization of CSF/plasma p-tau217, GFAP, sTREM2, and IRS-1 phosphorylation panels alongside CNS-penetrating DAT-SPECT or FDG-PET imaging to establish validated, cross-trial response biomarkers.

## 9 Challenges, limitations, and unresolved questions

Despite a strong mechanistic rationale and encouraging signals in early-phase trials, several substantial challenges continue to limit the translation of GLP-1 RAs into approved disease-modifying therapies for PD and AD.

A central open question is whether observed clinical effects reflect direct neuroprotection or an indirect consequence of improved systemic metabolism (Hölscher, 2024). Beyond glucose lowering, GLP-1 RAs produce weight reduction, improved insulin sensitivity, systemic anti-inflammatory effects, and cardiovascular benefit, any of which could independently influence neurological outcomes (Hong et al., 2024; Marso et al., 2016a; Wilding et al., 2021). Part of the epidemiological association between GLP-1 RA use and lower dementia incidence described in Section 1 may plausibly be mediated, at least in part, through these established cardioprotective and cerebrovascular effects (Section 4.5) rather than through direct neuronal action; reduced vascular disease burden is itself a recognized risk factor for lower dementia incidence, and current trial and cohort study designs are generally not able to fully separate this indirect vascular pathway from direct neuroprotection. Although direct stimulation of neuronal GLP-1 receptors is well established in experimental systems, disentangling the relative contribution of direct and indirect mechanisms in clinical practice, particularly among patients with comorbid diabetes, remains difficult (Hölscher, 2024).

Patient heterogeneity presents a further interpretive challenge. Although accumulating evidence implicates insulin resistance in neurodegeneration, it remains uncertain whether GLP-1 RAs confer comparable benefit in patients with PD or AD who lack insulin resistance (Hölscher, 2024). Methodological limitations compound this uncertainty: completed trials have generally been small and of limited duration relative to the timescales over which disease-modifying effects would be expected to manifest (Athauda et al., 2017). This mismatch between trial duration and expected effect latency deserves particular emphasis. Neurodegenerative pathology accumulates over years to decades, yet the completed PD trials have run for 48–96 weeks and the AD trials for up to 156 weeks; a treatment effect that requires a longer interval to become clinically detectable – for example, because it acts by slowing, rather than reversing, an ongoing degenerative process – could plausibly be missed by a trial of this length, particularly in AD, where the EVOKE/EVOKE+ null result cannot fully exclude a small effect that would only diverge from placebo beyond the 104-week primary assessment window.

Placebo response further complicates interpretation, particularly in PD. In the LIXIPARK trial, the placebo group’s MDS-UPDRS Part III score worsened by a mean of 3.04 points over 12 months (Section 5.3), a magnitude comparable to the treatment effect itself in several other PD trials; a similarly sized placebo trajectory could obscure a genuine but modest treatment effect in an inadequately powered study, or conversely could be mistaken for genuine disease progression against which an apparent treatment benefit is measured. In PD specifically, placebo response and variability in motor assessment can obscure modest treatment effects, while the inherently slow progression of AD makes demonstrating neuroprotection particularly difficult using conventional cognitive endpoints (Cummings et al., 2024; Silbert et al., 2024).

Statistical power for detecting modest effects, and for supporting reliable subgroup analyses, is a related and largely separate concern from trial duration. The Exenatide-PD3 confidence interval for the primary endpoint (95% CI −1.56 to 3.39) was wide enough to be compatible with a clinically modest true benefit despite the point estimate favoring no effect, illustrating that a single “negative” phase III trial does not definitively exclude a smaller effect than the phase II data suggested. Similarly, the three-arm, 99-participant MOST-ABLE trial (Section 5.4) is explicitly a dose-finding study and was not designed with the sample size needed for a definitive efficacy subgroup analysis. Across this literature more broadly, few trials have been powered *a priori* to detect differential treatment effects within pre-specified biological subgroups (for example, by baseline insulin resistance or CSF biomarker status), so that the subgroup hypotheses discussed in Section 8 remain largely exploratory and require confirmation in adequately powered, pre-specified analyses.

Incomplete characterisation of CNS drug exposure represents another substantial limitation. Although preclinical studies demonstrate blood–brain barrier penetration, human pharmacokinetic data, including cerebrospinal fluid exposure and direct evidence of CNS receptor engagement, remain limited, particularly for newer agents such as semaglutide (Holscher, 2024). Whether currently achievable CNS drug levels are sufficient for sustained receptor engagement and neuroprotection is unresolved and may partly explain the divergent results observed across individual GLP-1 RAs (Holscher, 2024).

Safety considerations acquire particular importance in elderly patients with neurodegenerative disease (Hong et al., 2024). Gastrointestinal adverse effects, including nausea, vomiting, and delayed gastric emptying, may pose a greater burden in PD, where autonomic dysfunction and reduced gastrointestinal motility already compromise oral levodopa absorption (Section 7) (Fasano et al., 2015; Rubino et al., 2021). Although the overall safety profile of GLP-1 RAs is favorable, ongoing monitoring for pancreatic, thyroid, and cardiovascular events, as well as for excessive weight loss in frail older patients, remains essential (Marso et al., 2016a; Marso et al., 2016b).

Finally, as a matter of interpretive discipline for this review itself: mechanistic findings are, in places, discussed collectively across the GLP-1 RA class for narrative clarity, but as Section 7 details, individual agents differ substantially in bioavailability, blood–brain barrier permeability, receptor selectivity, and formulation. Mechanistic or clinical findings established for one agent should not be assumed to generalize to others in this class without direct supporting evidence.

## 10 Future directions

Advancing GLP-1 RAs toward approved disease-modifying use in PD and AD will require addressing the specific limitations identified in completed and ongoing trials. Future research should prioritize earlier intervention, enrolling participants in the prodromal stage of disease, including individuals with REM sleep behavior disorder at risk for PD and those with preclinical or prodromal AD, when neuroprotective strategies are most likely to preserve neuronal function.

Trial design itself should evolve in several specific, concrete respects in response to the limitations discussed in Section “9 Challenges, limitations, and unresolved questions.” Treatment and follow-up durations should be extended beyond the 96- to 156-week windows used to date where feasible, particularly for AD, to allow a slowly accumulating disease-modifying effect to become statistically detectable against the background of a large placebo-treated cohort. Trials should be prospectively powered, not only for the primary endpoint but for pre-specified biological subgroup analyses (for example, by baseline insulin resistance, CSF p-tau status, or FDG-PET signature), so that the stratification hypotheses discussed in Section 8 can be formally tested rather than explored *post hoc*. Endpoint selection should also be reconsidered: composite imaging-and-fluid-biomarker endpoints, interpreted alongside rather than instead of clinical endpoints, may offer greater sensitivity to a genuine but modest disease-modifying effect than a single clinical scale, provided the biomarker–outcome dissociation discussed in Section 8 is explicitly accounted for in trial interpretation rather than treated as equivalent evidence of efficacy.

Advances in precision medicine will be equally important. Integrating fluid biomarkers, neuroimaging, and metabolic markers of insulin resistance into trial design may improve patient selection, identify individuals most likely to benefit, and provide objective measures of target engagement and disease progression. In parallel, a more complete understanding of CNS pharmacokinetics will be needed to guide development of brain-penetrant GLP-1 analogues with improved central bioavailability.

Future therapeutic strategies should also extend beyond GLP-1 monotherapy. Dual GLP-1/GIP agonism, triple GLP-1/GIP/glucagon agonism, and combination approaches pairing GLP-1 RAs with anti-amyloid or α-synuclein-targeted therapies may offer additional neuroprotective benefit by addressing complementary disease pathways. Large-scale real-world studies and continued pharmacovigilance will remain necessary to verify effectiveness and safety as these strategies advance toward clinical use.

## 11 Conclusion

GLP-1 receptor agonists represent a mechanistically plausible drug-repurposing strategy in PD and AD, engaging multiple pathways implicated in neurodegeneration, including neuroinflammation, mitochondrial dysfunction, insulin resistance, oxidative stress, and protein aggregation. Relative to conventional symptomatic therapies, this multi-target mode of action provides a biological rationale for continued investigation, though, as this review has emphasized throughout, the strength of that rationale currently differs markedly between PD, where two of three completed randomized trials showed a modest, washout-persistent benefit, and AD, where no completed randomized trial has yet met its primary endpoint (Section 6.4).

Despite promising preclinical and early-phase clinical data, definitive evidence of disease modification is still lacking in either disease. The outcomes of recent phase III trials underscore that translating mechanistic promise into confirmed clinical benefit is far from straightforward, and will likely require improved trial design, earlier intervention, biomarker-guided patient selection, adequately powered subgroup analyses, and strategies to increase CNS drug exposure (Sections 9, 10).

The negative or null results of the Exenatide-PD3, ELAD, and EVOKE/EVOKE+ phase III and IIb trials also carry direct regulatory implications: in their current form, none of these datasets would be expected to support a disease-modification indication, and sponsors and regulators will need to determine whether the underlying hypothesis merits further, differently designed confirmatory trials or whether it should be set aside in favor of other candidate mechanisms, a decision this review does not attempt to prejudge.

At present, the available evidence does not support off-label use of GLP-1 RAs for neurodegenerative disease outside the context of clinical trials, a conclusion that applies irrespective of whether an individual patient has comorbid diabetes, since no trial to date has demonstrated a neurodegeneration-specific benefit that would justify use for this indication alone. GLP-1 RAs remain one of several biologically plausible candidate strategies currently under investigation for disease modification in PD and AD, alongside other mechanistically distinct approaches under active development; continued, biomarker-informed trials, designed with the specific limitations identified in this review in mind, are warranted to determine whether metabolic modulation can be translated into meaningful neuroprotection.
